# Optimization of Culture Conditions for Production of the Anti-Leukemic Glutaminase Free L-Asparaginase by Newly Isolated *Streptomyces olivaceus* NEAE-119 Using Response Surface Methodology

**DOI:** 10.1155/2015/627031

**Published:** 2015-06-09

**Authors:** Noura El-Ahmady El-Naggar, Hassan Moawad, Nancy M. El-Shweihy, Sara M. El-Ewasy

**Affiliations:** ^1^Department of Bioprocess Development, Genetic Engineering and Biotechnology Research Institute, City of Scientific Research and Technological Applications, Alexandria 21934, Egypt; ^2^Department of Agricultural Microbiology, National Research Center, Cairo, Egypt

## Abstract

Among the antitumor drugs, bacterial enzyme L-asparaginase has been employed as the most effective chemotherapeutic agent in pediatric oncotherapy especially for acute lymphoblastic leukemia. Glutaminase free L-asparaginase producing actinomycetes were isolated from soil samples collected from Egypt. Among them, a potential culture, strain NEAE-119, was selected and identified on the basis of morphological, cultural, physiological, and biochemical properties together with 16S rRNA sequence as *Streptomyces olivaceus* NEAE-119 and sequencing product (1509 bp) was deposited in the GenBank database under accession number KJ200342. The optimization of different process parameters for L-asparaginase production by *Streptomyces olivaceus* NEAE-119 using Plackett-Burman experimental design and response surface methodology was carried out. Fifteen variables (temperature, pH, incubation time, inoculum size, inoculum age, agitation speed, dextrose, starch, L-asparagine, KNO_3_, yeast extract, K_2_HPO_4_, MgSO_4_·7H_2_O, NaCl, and FeSO_4_·7H_2_O) were screened using Plackett-Burman experimental design. The most positive significant independent variables affecting enzyme production (temperature, inoculum age, and agitation speed) were further optimized by the face-centered central composite design-response surface methodology.

## 1. Introduction

L-asparaginase (L-asparagine aminohydrolase, EC 3.5.1.1) is an important enzyme as therapeutic agents used in combination therapy with other drugs in the treatment of acute lymphoblastic leukemia in children, Hodgkin disease, acute myelocytic leukemia, acute myelomonocytic leukemia, chronic lymphocytic leukemia, lymphosarcoma treatment, reticulosarcoma, and melanosarcoma [1, 2]. L-asparaginase has an antioxidant property [3]. It is also used in food industry as a food processing aid; it can effectively reduce the level of acrylamide up to 90% in a range of starchy fried foods without changing the taste and appearance of the end product [4].

Notwithstanding its high therapeutic efficacy, the therapeutic use of L-asparaginase by the patients exerts toxicity to normal cells which in turn causes the unpleasant side effects to the patients. L-asparaginase administration has been limited by a high rate of hypersensitivity in the long-term use [5] and development of anti-asparaginase antibodies, which causes an anaphylactic shock or neutralization of the drug effect. To overcome these limitations, modified versions of L-asparaginase (such as L-asparaginase from other new sources, pegylated formulations, and L-asparaginase loaded into erythrocytes) have been recently proposed [6]. The L-asparaginases of* Erwinia chrysanthemi *and* E. coli *have been employed for many years as effective drugs in the treatment of acute lymphoblastic leukaemia and leukaemia lymphosarcoma [7], but their therapeutic response rarely occurs without some evidence of toxicity [8], suggesting the need to discover new L-asparaginases that are serologically different but have similar therapeutic effects. Therefore there is a continuing need to screen newer organisms in order to obtain strains capable of producing new and high yield of L-asparaginase with less adverse effects [9].

The toxicity of L-asparaginases is partially attributable to the glutaminase activity of these enzymes [10]. L-glutamine is required for several metabolic pathways including the formation of L-asparagine by the enzyme L-asparagine synthetase [11]. The L-glutaminase activity may cause such a reduction in glutamine in the body that it limits the tolerable therapeutic dose. Nowadays most of the research is focused on production of glutaminase free L-asparaginase by using microbial systems. L-asparaginases with high specificity for L-asparagine and low-to-negligible activity against L-glutamine are reported to be less troublesome during the course of anticancer therapy [12].

Microorganisms like bacteria, fungi, yeast, actinomycetes, and algae are very efficient producers and the better source of L-asparaginase, because they can be cultured easily and the extraction and the purification of L-asparaginase from them are also convenient, facilitating the large scale production [13].

Most of the microbial L-asparaginase is intracellular in nature except few which are secreted outside the cell [14]. Extracellular L-asparaginase is more advantageous than intracellular type because of higher accumulation of enzyme in culture broth under normal conditions, easy extraction, and downstream processing [15]; the extracellular L-asparaginase in bacteria is protease deficient and the liberated protein exported to the medium is mostly soluble and biologically active and has an authentic N-terminus, relatively free from endotoxins that lead to the minimization of adverse effects. Secretion also facilitates proper folding of proteins specially that requiring disulfide bridge formation, as it passes through a more favorable redox potential in the periplasmic space [16].

Production of L-asparaginase is greatly influenced by fermentation medium composition and culture conditions such as temperature, pH, inoculum size, agitation rate, and incubation time [17]. Statistical experimental designs have been used for many decades by several researchers in biotechnology for an optimization strategy [18–21] and can be adopted on several steps, the first step is to screen the important parameters and the second step is to optimize those parameters [22]. These have several advantages that included less experiment numbers, suitability for multiple factor experiments, search for relativity between factors, and finding of the most suitable conditions and forecast response [23]. Response surface methodology (RSM) is an efficient strategic experimental tool by which the optimal conditions of a multivariable system can be determined.

In the present study, strain NEAE-119 was identified as* Streptomyces olivaceus* strain NEAE-119. A statistical approach has been employed for which a Plackett-Burman design is used for identifying significant variables influencing glutaminase free L-asparaginase production by* Streptomyces olivaceus* NEAE-119. The levels of the positive significant variables were further optimized using face-centered central composite design.

## 2. Materials and Methods

### 2.1. Microorganisms and Cultural Conditions

Actinomycete strains used in this study were isolated from various soil samples collected from different localities in Egypt. Actinomycetes from the soils had been isolated using standard dilution plate method procedure on Petri plates containing starch nitrate agar medium of the following composition (g/L): starch, 20; KNO_3_, 2; K_2_HPO_4_, 1; MgSO_4_·7H_2_O, 0.5; NaCl, 0.5; CaCO_3_, 3; FeSO_4_·7H_2_O, 0.01; agar, 20, and distilled water up to 1 L; then plates were incubated for a period of 7 days at 30°C.* Streptomyces* isolates were purified and maintained as spore suspensions in 20% (v/v) glycerol at −20°C for subsequent investigation.

### 2.2. Screening of L-Asparaginase Production by Plate Assay

It is generally observed that L-asparaginase production is accompanied by an increase in pH of the culture filtrates [24]. The plate assay was based on Gulati et al. [25] method with the incorporation of pH indicator phenol red (prepared in ethanol) in medium containing L-asparagine (sole nitrogen source). Phenol red at acidic pH is yellow and at alkaline pH turns pink; thus a pink zone is formed around microbial colonies producing L-asparaginase. Screening of potential L-asparaginase producing actinomycetes was carried out with the use of asparagine dextrose salts agar (ADS agar) (g/L: asparagine 10, dextrose 2, K_2_HPO_4_ 1, MgSO_4_ 0.5, and agar 20); pH was adjusted to 6.8 and supplemented with phenol red as a pH indicator (0.009% final concentration) [25] and sterilized at 1.5 atmospheric pressure for 20 min. Inoculated plates were incubated at 30°C for 7 days. Plates were examined for change in color of medium from yellowish to pink due to change of pH indicating the positive asparaginase activity. Colonies with pink zones were considered as L-asparaginase producing strains. Isolates exhibiting L-asparaginase activity were selected for further study. Control plates were prepared as inoculated medium without dye and uninoculated medium with dye.

### 2.3. Agar Well Diffusion Technique

L-asparaginase-producing strains were selected for subsequent screening under submerged fermentation conditions. Fifty mL of asparagine dextrose salts broth medium were dispensed in 250 mL Erlenmeyer conical flasks, sterilized, inoculated, and incubated at 30°C for 5 days in a rotatory incubating shaker at 150 rpm. After the incubation time, the mycelium of each isolate was collected by centrifugation at 6000 rpm for 20 min. 100 *μ*L of cell free culture broth was poured into the agar well of diameter 8 mm prepared in plates containing asparagine dextrose salts agar medium supplemented with phenol red. The filtrate was allowed to diffuse into the medium for 12 hours at 4°C. The diameter of zone (mm) of L-asparaginase activity, as indicated by the formation of pink colored zone around the well against the yellow background, was measured. For further studies, cultures showing greater enzyme production were selected.

### 2.4. Inoculum Preparation

250 mL Erlenmeyer flasks containing 50 mL of asparagine dextrose salts broth (g/L: asparagine 10, dextrose 2, K_2_HPO_4_ 1, and MgSO_4_ 0.5) were inoculated with three disks of 8 mm diameter taken from the 7-day-old stock culture grown on starch nitrate agar medium. The flasks were incubated for 48–72 h in a rotatory incubator shaker at 30°C and 150 rpm and were used as inoculum for subsequent experiments.

### 2.5. Production of L-Asparaginase by Submerged Fermentation

The selected strain was cultured in fifty mL of asparagine dextrose salts broth medium (at a specified pH) dispensed in 250 mL Erlenmeyer conical flasks. The inoculated flasks were incubated on a rotatory incubator shaker at 30–37°C with shaking at 100–200 rpm. After the specified incubation time for each set of experimental trials, the mycelium of the tested isolate was collected by centrifugation at 5000 g for 20 min at 4°C.

### 2.6. Assay of L-Asparaginase Activity

L-asparaginase activity was determined by measuring the amount of ammonia formed by nesslerization [26]. The reaction mixture contains 1.5 mL of 0.04 M L-asparagine prepared in 0.05 M Tris-HCl buffer, pH 8.6, and 0.5 mL of an enzyme to make up the total volume to 2 mL. The tubes were incubated at 37°C for 30 minutes. The reaction was stopped by adding 0.5 mL of 1.5 M Trichloroacetic acid (TCA). The blank was prepared by adding enzyme after the addition of TCA. The precipitated protein was removed by centrifugation at 10,000 g for 5 min and the liberated ammonia in the supernatant was determined colorimetrically by direct nesslerization by adding 1 mL Nessler's reagent into tubes containing 0.5 mL of clear supernatant and 7 mL of distilled water and incubated at room temperature for 20 min. A yellow coloration indicates the presence of ammonia: at higher concentrations, a brown precipitate may form. The yellow color was read using a UV-visible spectrophotometer (Optizen Pop—UV/Vis spectrophotometer) at 480 nm. The amount of ammonia liberated was calculated using ammonium chloride standard curve. One unit (U) of L-asparaginase is defined as the amount of enzyme which catalyzed the formation of 1 *µ*mole of ammonia from L-asparagine per minute under the standard assay conditions.

### 2.7. Assay of L-Glutaminase

L-Glutaminase activity was determined using L-glutamine as substrate and the product ammonia, released during the catalysis, was measured by using Nessler's reagent. L-glutaminase was assayed according to Imada et al. [27].

### 2.8. Morphology and Cultural Characteristics

The spore chain morphology, the spore surface ornamentation, and spore size of strain NEAE-119 were examined on starch nitrate agar medium after 14 days at 30°C. The gold-coated dehydrated specimen was examined with Analytical Scanning Electron Microscope Jeol JSM-6360 LA operating at 20 Kv at the Central Laboratory, City of Scientific Research and Technological Applications, Alexandria, Egypt. Aerial spore-mass color, substrate mycelial pigmentation, and the production of diffusible pigments were observed on ISP media (1–7) as described by Shirling and Gottlieb [28].

### 2.9. Chemotaxonomy and Physiological Characteristics

Sugars and diaminopimelic acid (DAP) isomers were identified by the method described by Staneck and Roberts [29]. Physiological characteristics were performed following the methods of Shirling and Gottlieb [28]. The ability of the organism to inhibit the growth of several bacterial, yeast, and fungal strains was determined: three bacterial strains (*Staphylococcus aureus *A9897,* Pseudomonas aeruginosa* T9934, and* Klebsiella pneumonia* A9898) isolated from various clinical specimens and kindly provided by Infection Control Unit, Department of Medical Microbiology and Immunology, Faculty of Medicine, Mansoura University, Mansoura, Egypt and two bacterial strains belonging to the Culture Collection of NRRL: Gram-positive (*Bacillus subtilis *NRRL B-543) and Gram-negative (*Escherichia coli *NRRL B-210), yeast (*Candida albicans* NRRL Y-477), and five fungal strains (*Rhizoctonia solani, Fusarium oxysporum, Alternaria solani, Bipolaris oryzae,* and* Aspergillus niger*) kindly provided by Plant Pathology Department, Faculty of Agriculture, Mansoura University, Egypt. Some additional tests can be considered to be useful in completing the description of a strain or species, even if they are not very significant or indicative on their own. The ability of strain NEAE-119 to produce uricase [30] and asparaginase activity [25] was tested.

### 2.10. 16S rRNA Sequencing

The preparation of genomic DNA of the strain was performed according the method described by Sambrook et al. [31]. The PCR reaction was performed according to the method of El-Naggar et al. [32].

### 2.11. Sequence Alignment and Phylogenetic Analysis

The complete 16S rRNA gene sequence (1509 bp) of strain NEAE-119 was aligned with the corresponding 16S rRNA sequences of the type strains of representative members of the genus* Streptomyces* retrieved from the GenBank, EMBL, DDBJ, and PDB databases by using BLAST program (http://blast.ncbi.nlm.nih.gov/Blast.cgi) [33] and the software package MEGA4 version 2.1 [34] was used for multiple alignment and phylogenetic analysis. The phylogenetic tree was constructed via the bootstrap test of neighbor-joining algorithm [35] based on the 16S rRNA gene sequences of strain NEAE-119 and related organisms.

### 2.12. Selection of Significant Variables by Plackett-Burman Design

The Plackett-Burman statistical experimental design [36] is a two factorial design, which identifies the critical physicochemical parameters required for elevated production, and is very useful for screening the most important factors with respect to their main effects [37]. This model does not describe interaction among factors and it is used to screen and evaluate the important factors that influence the response. The total number of experiments to be carried out according to Plackett-Burman is *n* + 1, where *n* is the number of variables. Sixteen different independent variables including temperature, pH, incubation time, inoculum size, inoculum age, agitation speed, dextrose, starch, L-asparagine, KNO_3_, yeast extract, K_2_HPO_4_, MgSO_4_·7H_2_O, NaCl, and FeSO_4_·7H_2_O were screened in Plackett-Burman experimental design. Each variable is represented at two levels, high and low, denoted by (+) and (−), respectively (Table 1). Plackett-Burman experimental design is based on the first order model:

(1) $$\begin{array}{c} Y=\beta_{0}+\overset{}{\underset{}{\sum }}\beta_{i}X_{i}, \end{array}$$

where *Y* is the response or dependent variable (L-asparaginase activity) and it will always be the variable we aim to predict, *β*_0_ is the model intercept and *β*_*i*_ is the linear coefficient, and *X*_*i*_ is the level of the independent variables; it is the variables that will help us to explain L-asparaginase activity. All trials were performed in duplicate and the average of L-asparaginase activity was treated as responses.

### 2.13. Face-Centered Central Composite Design (FCCD)

This step involved optimization of the levels and the interaction effects between various significant variables which exerted a positive effect on the L-asparaginase activity by using face-centered central composite design (FCCD). FCCD is an effective design that is used for sequential experimentation and provides reasonable amount of information for testing the goodness of fit and does not require large number of design points thereby reducing the overall cost associated with the experiment [38]. In this study, the experimental plan consisted of 20 trials and the independent variables were studied at three different levels, low (−1), middle (0), and high (+1). The center point was repeated six times in order to evaluate the curvature and the experiment replication facilitated the pure error estimation, so that the significant lack of fit of the models could be predicted. All the experiments were done in duplicate and the average of L-asparaginase activity obtained was taken as the dependent variable or response (*Y*). The experimental results of FCCD were fitted via the response surface regression procedure using the following second order polynomial equation:

(2) $$\begin{array}{c} Y=\beta_{0}+\overset{}{\underset{i}{\sum }}\beta_{i}X_{i}+\overset{}{\underset{ii}{\sum }}\beta_{ii}X_{i}^{2}+\overset{}{\underset{ij}{\sum }}\beta_{ij}X_{i}X_{j}, \end{array}$$

in which *Y* is the predicted response, *β*_0_ is the regression coefficients, *β*_*i*_ is the linear coefficient, *β*_*ii*_ is the quadratic coefficients, *β*_*ij*_ is the interaction coefficients, and *X*_*i*_ is the coded levels of independent variables. However, in this study, the independent variables were coded as *X*_1_, *X*_5_, and *X*_6_. Thus, the second order polynomial equation can be presented as follows:

(3) Y=β0+β1x1+β5x5+β6x6+β15x1x5+β16x1x6 +β56x5x6+β11x12+β55x52+β66x62.

### 2.14. Statistical Analysis

The experimental data obtained was subjected to multiple linear regressions using Microsoft Excel 2007. The *P* values were used as a tool to check the significance of the interaction effects, which in turn may indicate the patterns of the interactions among the variables [39]. The statistical software package, STATISTICA software (Version 8.0, StatSoft Inc., Tulsa, USA), was used to plot the three-dimensional surface plots.

## 3. Results and Discussion

**Figure 1:**
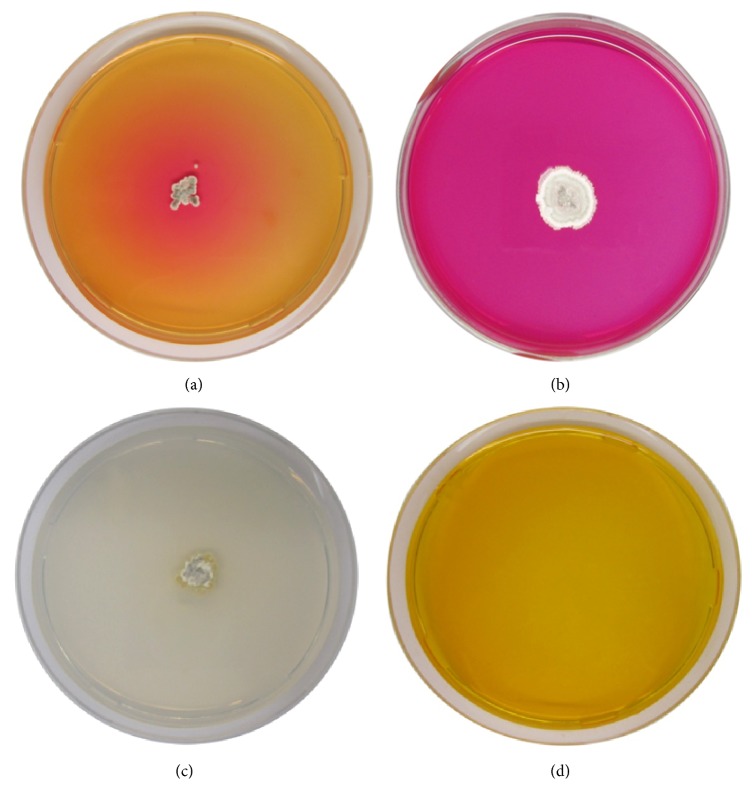
L-asparaginase activity of* Streptomyces *sp. NEAE-119 detected by plate assay. (a, b) Production of the enzyme indicated by color change in the medium from yellow to pink zone surrounding the colony after two and five days, respectively. (c) Control plates were prepared as inoculated medium without dye. (d) Uninoculated medium with dye.

L-asparaginase activity of* Streptomyces *sp. NEAE-119 was detected by plate assay. Production of the enzyme was indicated by color change in the medium from yellow to pink zone surrounding the colony (Figure 1). L-asparaginase activity was confirmed by agar-well diffusion technique. The potential culture, strain NEAE-119, was identified on the basis of morphological, cultural, physiological, and chemotaxonomic properties, together with 16S rRNA sequence as* Streptomyces olivaceus* strain NEAE-119.

### 3.1. Morphology and Cultural Characteristics of Isolate Number NEAE-119

**Figure 2:**
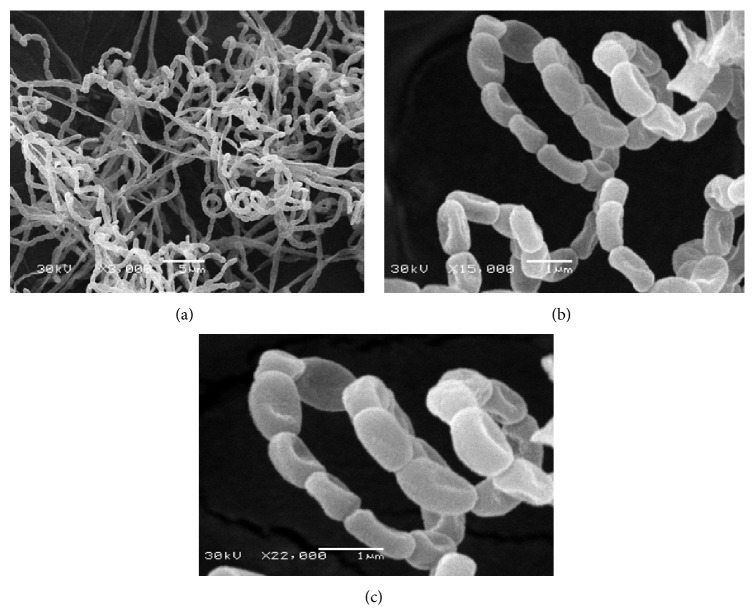
Scanning electron micrograph showing the spore chain morphology and spore surface ornamentation of strain NEAE-119 grown on starch nitrate agar medium for 14 days at 30°C at magnification of 3000x (a), 15000x (b), and 22000x (c).

Morphological observation of the 14-day-old culture of strain NEAE-119 grown on yeast extract-malt extract agar (ISP 2) [28] revealed that strain NEAE-119 had the typical characteristics of the genus* Streptomyces *[40]; it is aerobic and mesophilic; both aerial and vegetative hyphae were abundant, well-developed, and not fragmented. Aerial mycelium color was varied from the grey color to greyish beige or whitish grey on different test media. Cultural characteristics of strain NEAE-119 are shown in the table in the Supplementary Materials available online at http://dx.doi.org/10.1155/2015/627031. Strain NEAE-119 grew well on yeast extract-malt extract agar (ISP medium 2), oatmeal agar (ISP medium 3), inorganic salt-starch agar (ISP medium 4), glycerol-asparagine agar (ISP medium 5), peptone-yeast extract iron agar (ISP medium 6), and tyrosine agar (ISP medium 7). Verticils are not present. The mycelium does not fragment. It formed an extensively branched substrate mycelium and aerial hyphae which differentiated into spore chains. Spore chains with many spores were in section* Spirals*, with open spirals intergrading through flexuous spore chains suggestive of section *Rectiflexibiles*. Mature spore chains are generally long, often with more than 50 spores per chain. This morphology is seen on starch nitrate agar medium. Spore surface is smooth (0.55–0.90 × 1.16–1.34 *μ*m in diameter) (Figure 2).

### 3.2. Physiological and Biochemical Characteristics

The physiological characteristics of strain NEAE-119 are shown in Table 2. Strain NEAE-119 grew well on yeast extract-malt extract agar (ISP medium 2). The substrate hyphae are yellowish grey; substrate mycelium pigment is not a pH indicator. No pigment was found in medium in yeast extract-malt extract agar. Melanoid pigments were not formed in peptone-yeast-iron agar and tyrosine agar. Starch hydrolysis, lecithinase activity, milk coagulation and peptonization, growth on cellulose, and nitrate reduction were positive. Gelatin liquification, melanin production, and hydrogen sulphide production were negative. *α*-amylase, cellulase, uricase, chitosanase, and asparaginase are produced while protease is not produced. D-fructose, D-xylose, D-galactose, D-Glucose, L-arabinose, ribose, D-mannose, sucrose, maltose, rhamnose, cellulose, and trehalose are utilized for growth. It exhibited antimicrobial activity against* Staphylococcus aureus, Alternaria solani, *and* Bipolaris oryzae.* It exhibited no antimicrobial activity against* Candida albicans, Bacillus subtilis, Escherichia coli, Pseudomonas aeruginosa, Klebsiella pneumonia, Rhizoctonia solani, Fusarium oxysporum,* and* Aspergillus niger.* The optimal growth temperature of strain NEAE-119 was 30°C and optimal pH was 7.0. Data for reference species (*Streptomyces olivaceus*) were taken from Bergey's Manual of Systematic Bacteriology: Volume 5: the Actinobacteria [40].

Chemotaxonomic tests showed that the cell wall contained LL-diaminopimelic acid in whole-organism hydrolysates, indicating that it was of cell-wall type I. The whole-cell hydrolysates contained mainly mannose and arabinose. On the basis of morphological, cultural, and chemotaxonomic properties, together with the physiological properties of strain NEAE-119 shown in Table 2, it is evident that strain NEAE-119 belongs to the genus* Streptomyces.*

### 3.3. 16S rRNA Gene Sequence Comparisons and Phylogenetic Analysis

**Figure 3:**
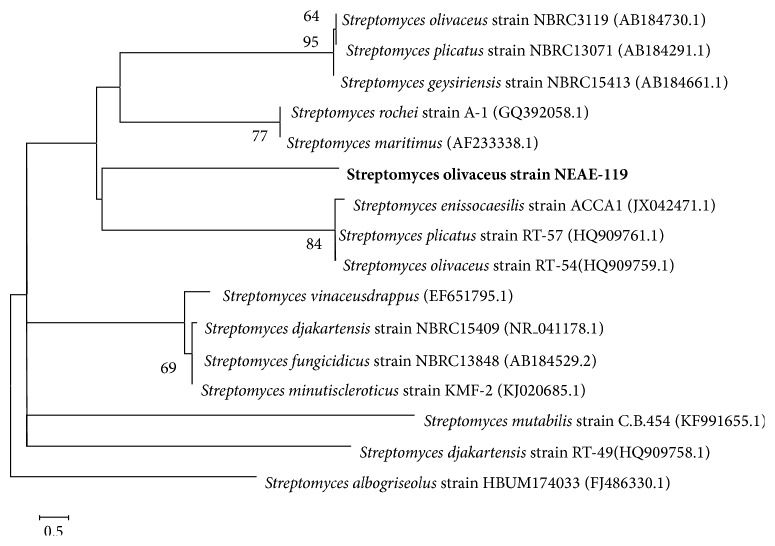
The phylogenetic tree was constructed via the bootstrap test of neighbor-joining algorithm based on the 16S rRNA gene sequences of strain NEAE-119 and related species of the genus* Streptomyces*. Only bootstrap values above 50%, expressed as percentages of 1000 replications, are shown at the branch points. GenBank sequence accession numbers are indicated in parentheses after the strain names. Phylogenetic analyses were conducted in the software package MEGA4. Bar: 0.5 substitution per nucleotide position.

The 16S rRNA gene sequence (1509 bp) was determined for strain NEAE-119. A BLAST search [33] of the GenBank database using this sequence showed its similarity to that of many species of the genus* Streptomyces*. A phylogenetic tree (Figure 3) based on 16S rRNA gene sequences of members of the genus* Streptomyces* was constructed according to the bootstrap test of neighbor-joining algorithm method of Saitou and Nei [35] with MEGA4 [34]. This tree shows the close phylogenetic association of strain NEAE-119 with certain other* Streptomyces *species. Phylogenetic analysis indicated that the strain NEAE-119 consistently falls into a clade together with* Streptomyces enissocaesilis* strain ACCA1 (GenBank/EMBL/DDBJ accession number JX042471.1, 99% sequence similarity),* Streptomyces plicatus* strain RT-57 (GenBank/EMBL/DDBJ accession number HQ909761.1, 99% sequence similarity), and* Streptomyces olivaceus* strain RT-54 (GenBank/EMBL/DDBJ accession number HQ909759.1, 99% sequence similarity). On the basis of the collected data and in view of the comparative study of the recorded properties of isolate number NEAE-119 in relation to the closest related species of the genus* Streptomyces*, it is most closely related to the type strains of* Streptomyces olivaceus* strain RT-54 (GenBank/EMBL/DDBJ accession number HQ909759.1) (99% sequence similarity). Therefore, this strain was identified as* Streptomyces olivaceus* strain NEAE-119 and its sequencing product was deposited in the GenBank database under accession number KJ200342.

### 3.4. Evaluation of Variables Affecting L-Asparaginase Activity Using Plackett-Burman Design

**Figure 4:**
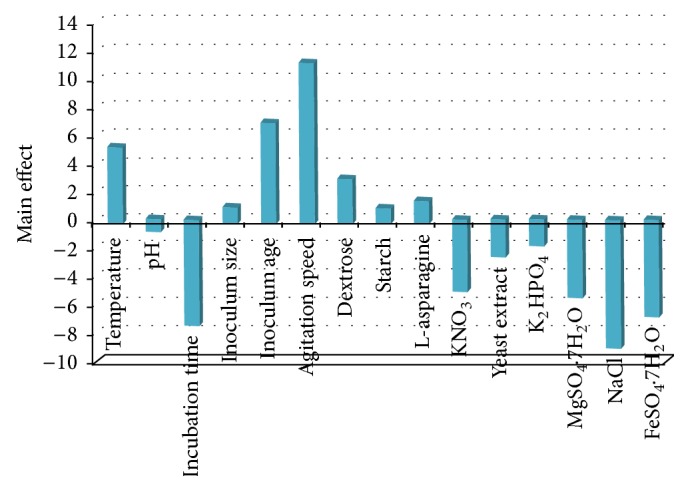
The main effects of the fermentation conditions on L-asparaginase production according to the Packett-Burman experimental results.

**Figure 5:**
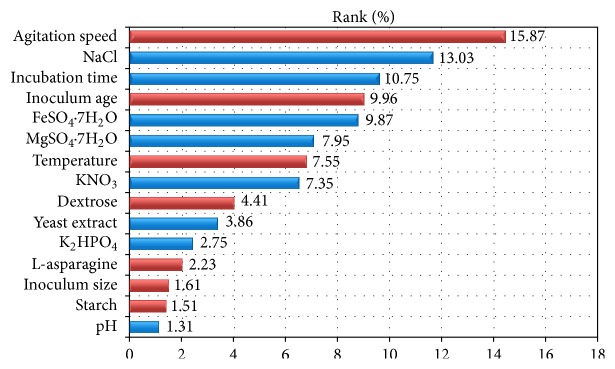
Pareto chart illustrates the order of significance of the variables affecting L-asparaginase production by* Streptomyces *sp. strain NEAE-119 (the red color represents positive effects and the blue color represents negative effects; ranks (%) values ranging from 1.31 to 15.87).

Sixteen different independent (assigned) variables including temperature, pH, incubation time, inoculum size, inoculum age, agitation speed, dextrose, starch, L-asparagine, KNO_3_, yeast extract, K_2_HPO_4_, MgSO_4_·7H_2_O, NaCl, and FeSO_4_·7H_2_O and four unassigned variables (commonly referred to as dummy variables) were screened in Plackett-Burman experimental design of 20 trials to study the effect of the selected variables on the production of L-asparaginase. Four dummy variables are used to estimate experimental errors in data analysis (Table 3). Table 3 represents the results of the screening of significant variables for L-asparaginase production and the corresponding response (*Y*) using Plackett-Burman design. The maximum L-asparaginase activity (49.874 U/mL) was achieved in the run number 15, while the minimum L-asparaginase activity (5.181 U/mL) was observed in the run number 5. Statistical analysis of the L-asparaginase activity was performed and represented in Table 4. With respect to the main effect of each variable (Figure 4), we can see that seven variables from the fifteen named including temperature, inoculum size, inoculum age, agitation speed, dextrose, starch and L-asparagine positively affect L-asparaginase production where the other eight variables named pH, incubation time, KNO_3_, yeast extract, K_2_HPO_4_, MgSO_4_·7H_2_O, NaCl, and FeSO_4_·7H_2_O negatively affect L-asparaginase production. The Pareto chart illustrates the order of significance of the variables affecting L-asparaginase production in Plackett-Burman experimental design (Figure 5). Among the fifteen variables, agitation speed showed the highest positive effect by 15.87%, followed by inoculum age (9.96%), and then temperature by 7.55%. Among the 15 variables, NaCl showed the highest negative significance by 13.03%. Next to NaCl, incubation time showed negative effect by 10.75% followed by FeSO_4_·7H_2_O and MgSO_4_·7H_2_O by 9.87% and 7.95%, respectively.

The *R* ^2^ values provide a measure of how much variability in the observed response values can be explained by the experimental factors. The *R* ^2^ value is always between 0 and 1. The closer *R* ^2^ is to the 1, the stronger the model is and the better it predicts the response [41]. In this case, the value of the determination coefficient (*R* ^2^ = 0.9791) indicates that 97.91% of the variability in the response was attributed to the given independent variables and only 2.09% of the total variations are not explained by the independent variables. In addition, the value of the adjusted determination coefficient (Adj. *R* ^2^ = 0.9010) is also very high which indicates a high significance of the model. A higher value of the correlation coefficient (*R* = 0.9895) signifies an excellent correlation between the independent variables, this indicated a good correlation between the experimental and predicted values. Thus, the analysis of the response trend using the model was considered to be reasonable. The significance of each coefficient was determined by Student's *t*-test and *P* values, which are listed in Table 4. The larger the magnitude of the *t*-value is and the smaller the *P* value is, the more significant the corresponding coefficient is [42]. In the current experiment, variables evidencing *P* values of less than 0.05 (confidence levels exceeding 95%) were considered to have significant effects on the L-asparaginase activity.

Agitation speed, with a probability value of 0.0022, *t*-value of 7.0197, and confidence level of 99.783, was determined to be the most significant factor, followed by NaCl (*P* value 0.0045, *t*-value −5.7627, and confidence level 99.550), incubation time (*P* value 0.0089, *t*-value −4.7554, and confidence level 99.106), inoculum age (*P* value 0.0116, *t*-value 4.4049, and confidence level 98.835), FeSO_4_·7H_2_O (*P* value 0.0120, *t*-value −4.3669, and confidence level 98.800), MgSO_4_·7H_2_O (*P* value 0.0245, *t*-value −3.5180, and confidence level 97.551), temperature (*P* value 0.0289, *t*-value 3.3382, and confidence level 97.111), and then KNO_3_ (*P* value 0.0314, *t*-value −3.2499, and confidence level 96.862). Screened significant variables, temperature, inoculum age, and agitation speed exerted positive effect on L-asparaginase production by* Streptomyces *sp. NEAE-119, whereas incubation time, KNO_3_, MgSO_4_·7H_2_O, NaCl, and FeSO_4_·7H_2_O exerted negative effect. On the basis of the calculated *t*-values (Table 4), temperature (*X*_1_), inoculum age (*X*_5_), and agitation speed (*X*_6_) were chosen for further optimization using FCCD, since these factors had the most positive effects on L-asparaginase production. The model *F* value of 12.5404 (Table 4) implies that the model is significant. The values of significance *F* (*P* value) < 0.05 (0.0126) indicate that model terms are significant. By neglecting the terms that were insignificant (*P* > 0.05), the first order polynomial equation was derived representing L-asparaginase production as a function of the independent variables:

(4) YL-asparaginase  production=16.196+2.642X1−3.763X3 +3.486X5+5.555X6 −2.572X10−2.784X13 −4.560X14−3.456X15,

where *Y* is the response (L-asparaginase production) and *X*_1_, *X*_3_, *X*_5_, *X*_6_, *X*_10_, *X*_13_, *X*_14_, and *X*_15_ are temperature, incubation time, inoculum age, agitation speed, KNO_3_, MgSO_4_·7H_2_O, NaCl, and FeSO_4_·7H_2_O, respectively.

**Figure 6:**
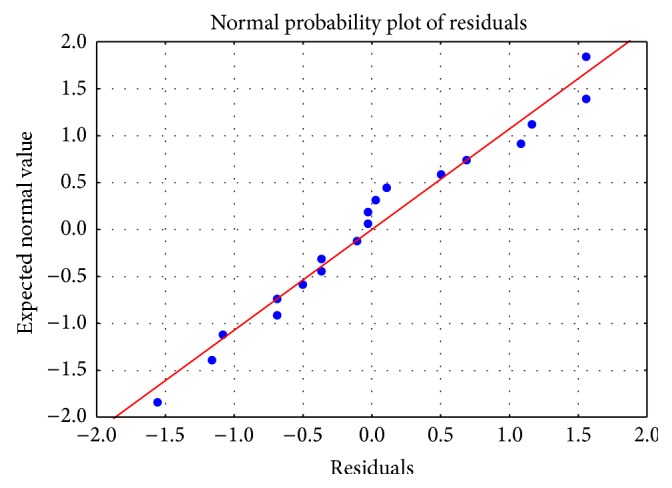
The normal probability plot of the residuals.

Checking the adequacy of the model needs all of the information on lack of fit, which is contained in the residuals. The normal probability plot of the residuals is an important diagnostic tool to detect and explain the systematic departures from the normality [39].  Figure 6 shows a plot of normal probability of the experimental results. The normal probability plot of the residuals shows the points close to a diagonal line; therefore, the residuals appear to be approximately normally distributed. This indicates that the model was well fitted with the experimental results.

Khamna et al. [43] have reported 30°C for the maximum activity and the growth by* Amycolatopsis *CMV-H002. Narayana et al. [14] have also reported that* Streptomyces albidoflavus *produces high amount of L-asparaginase at 35°C. Amena et al. [15] have reported that L-asparaginase activity was maximum at 40°C by* Streptomyces gulbargensis.* Siddalingeshwara and Lingappa [44] have reported 35°C as the optimum temperature for maximum L-asparaginase production. The temperature optima for L-asparaginase production vary widely in different strains. The variation may be due to the strains employed during the fermentation for L-asparaginase production and to fermentation conditions.

In the screening and fermentation development work which utilizes shaken cultures, it is essential that an oxygen level be provided which is sufficient to meet both the growth requirements of the organism and the yield of desired end product. Since aeration above or below an optimal level may induce conditions unsuitable for the formation of the desired end product, precise measurement and control of this variable are essential [45].

Since microorganisms growing in submerged culture utilize oxygen dissolved in the fermentation medium, the supply in vessels used for shaken cultures may become critical for microbial biosynthesis of specific end products. In addition, oxidation-reduction mechanisms existing in the fermentation mixture may exert a chemical influence on biosynthetic products [45]. Gentle aeration enables obtaining both good growth and high L-asparaginase yield [46]. Heinemann and Howard [47] observed that agitation in shaken culture was essential for optimal growth of* Serratia marcescens* and that the tumor inhibitory enzyme, asparaginase, was produced during a period of zero dissolved oxygen concentration in the fermentation medium.* Serratia marcescens* produces large amount of L-asparaginase with limited aeration than it does anaerobically [48]. Most of the organisms demand yeast extract for the growth and L-asparaginase production [49]. While using yeast extract, it was found to increase the viscosity of the medium, thereby reducing the oxygen uptake; good mixing was critical especially when fermentation medium becomes viscous [50]. In such cases, growth was limited by transfer of oxygen to the cell surface rather than by oxygen solubility which was observed in* E. coli* and* E. chrysanthemi* [51]. This suggests that lower level of aeration found to be suitable for the growth and yield of enzyme. This may be due to the fact that lower level of aeration may facilitate the suitable mycelium branching for the yield of enzyme.

Inoculum is generally transferred at the logarithmic phase of growth; the age of inoculum is important to achieve optimum yield of the metabolites. Inoculum age of 48–72 h was found to be the most suitable conditions for maximum production of L-asparaginase from* Streptomyces* sp. NEAE-119, because cells are in the logarithmic or early exponential growth phase and the cells are more active. The higher inoculum density is inhibitory to the enzyme production as too much biomass can deplete the substrate nutrients or accumulation of some nonvolatile self-inhibiting substances that inhibit the product formation [52] and lower density may give insufficient biomass causing induced product formation whereas higher inoculum may produce too much biomass which is inhibitory to the product formation [53]. Adequate inoculums can initiate fast mycelium growth and product formation, thereby reducing other organism contamination. Quantity of inoculum had a definite effect on enzyme titers.

According to Prakasham et al. [51] temperature and inoculum level are the major influential parameters and contributed to more than 50% of total L-asparaginase production. Amena et al. [15] have reported the inoculum size (1 × 10^8^ spores/mL) for the maximum L-asparaginase production of 6.9 IU/mL by* Streptomyces gulbargensis *using ground nut extract using submerged fermentation. Kumari et al. [54] have reported optimum inoculum level of 10.36% (v/v) for L-asparaginase production by *Streptomyces griseoluteus *WS3/1 under submerged fermentation.

### 3.5. Optimization by Face-Centered Central Composite Design

Face-centered central composite design was employed to study the optimal levels and the interactions among the selected significant factors; those had positive effect on the L-asparaginase production. The other variables in the study were maintained at a constant level which gave maximal yield in the Plackett-Burman experiments. In this study, a total of 20 experiments with different combination of temperature (*X*_1_), inoculum age (*X*_5_), and agitation speed (*X*_6_) were performed and the results of experiments for studying the effects of three independent variables on L-asparaginase activity are presented along with predicted response and residuals (Table 5). The results showed considerable variation in the L-asparaginase activity. Runs 1, 6, 10, 12, 14, and 15 showed a high L-asparaginase activity (≥67.403 U/mL). The minimum L-asparaginase activity (15.501 U/mL) was observed in run number 5, while the maximum L-asparaginase activity (70.076 U/mL) was achieved in run number 12.

### 3.6. Multiple Regression Analysis and ANOVA

Multiple regression analysis was used to analyze the data; the goodness of fit of the model was checked by the coefficient of determination (*R* ^2^), which was found to be 0.9503, indicating that the sample variation of 95.03% was attributed to the variables and only 4.97% of the total variance could not be explained by the model. Therefore, the present *R* ^2^-value reflected a very good fit between the observed and predicted responses and implied that the model is reliable for L-asparaginase production in the present study. Analysis of variance (ANOVA) which is required to test the significance and adequacy of the model is presented in Table 6. The analysis of variance (ANOVA) of the regression model demonstrates that the model is highly significant as is evident from Fisher's *F*-test (21.2771) and a very low probability value (2.22009*E* − 05). The significance of each coefficient was determined by *t*-values and *P* values which are listed in Table 6. The *P* values denote the significance of the coefficients and are also important in understanding the pattern of the mutual interactions between the variables. Interpretation of the data was based on the signs (positive or negative effect on the response) and statistical significance of coefficients (*P* < 0.05). Interactions between two factors could appear as an antagonistic effect (negative coefficient) or a synergistic effect (positive coefficient).

It can be seen from the degree of significance that the linear coefficients of inoculum age (*X*_5_), agitation speed (*X*_6_), interaction between temperature (*X*_1_), inoculum age (*X*_5_), and quadratic effect of temperature (*X*_1_), and agitation speed (*X*_6_) are significant. The probability values of the coefficient suggest that among the three variables studied, *X*_1_, *X*_5_ shows maximum interaction between the two variables (*P* value 0.0443), indicating that 95.57% of the model is affected by these variables. The linear coefficients of temperature (*X*_1_), interaction between *X*_1_ and *X*_6_, and quadratic effect of *X*_5_ are not significant (*P* value > 0.05). On the other hand, among the different interactions, interaction between *X*_1_ and *X*_6_ and that between *X*_5_ and *X*_6_ are not significant (*P* values 0.1690 and 0.4854, resp.), indicating that there is no significant correlation between each two variables and that they did not help much in increasing the production of L-asparaginase.

In order to evaluate the relationship between dependent and independent variables and to determine the maximum L-asparaginase production corresponding to the optimum levels of temperature (*X*_1_), inoculum age (*X*_5_), and agitation speed (*X*_6_), a second-order polynomial model (equation (5)) was proposed to calculate the optimum levels of these variables. By applying the multiple regression analysis on experimental data, the second-order polynomial equation that defines predicted response (*Y*) in terms of the independent variables (*X*_1_, *X*_5_, and *X*_6_) was obtained:

(5) YL-asparaginase  production=66.667−2.510X1+5.337X5 +10.927X6−4.495X1X5 −2.898X1X6−1.416X5X6 −10.073X12−4.923X52 −17.636X62,

where the *Y* is the predicted response, *X*_1_ the coded value of temperature, *X*_5_ the coded value of inoculum age, and *X*_6_ the coded value of agitation speed.

**Figure 7:**
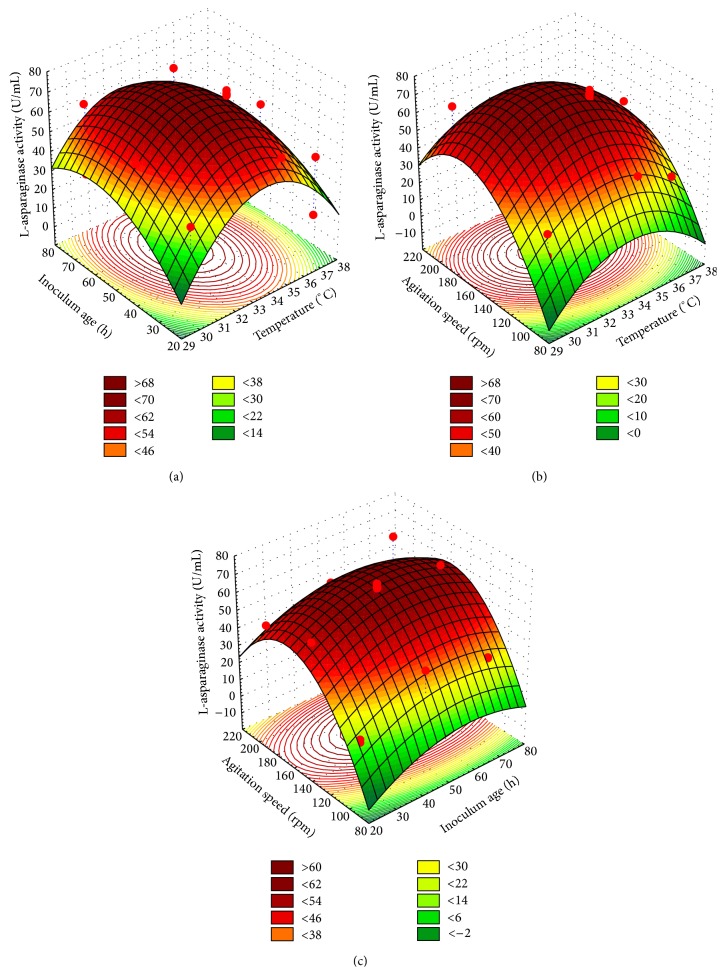
Three-dimensional response surface plots showing the effect of temperature (*X*_1_), inoculum age (*X*_5_) and agitation speed (*X*_6_) and their mutual effect on the production of L-asparaginase.

The interaction effects and optimal levels of the variables were determined by plotting the three-dimensional response surface curves (Figures 7(a)–7(c)) when one of the variables is fixed at optimum value and the other two are allowed to vary.  Figure 7(a) represents the L-asparaginase activity as a function of temperature (*X*_1_) and inoculum age (*X*_5_) by keeping agitation speed (*X*_6_) at optimum value. It showed that lower and higher levels of temperature support relatively low levels of L-asparaginase activity; the highest value of L-asparaginase activity was obtained with middle level of temperature and inoculum age. Further increase of inoculum age did not result in higher L-asparaginase activity.  Figure 7(b) represents the L-asparaginase activity as a function of temperature (*X*_1_) and agitation speed (*X*_6_) by keeping inoculum age (*X*_5_) at optimum value; the maximum L-asparaginase activity was attained at moderate to high levels of agitation speed and moderate levels of temperature and further increase in the temperature resulted in a gradual decrease in the L-asparaginase activity.  Figure 7(c) showed that the maximum L-asparaginase production was attained beyond middle levels of inoculum age and lower and higher levels of inoculum age resulted in a gradual decrease in L-asparaginase production. Highest value of L-asparaginase production was obtained beyond high agitation speed.

### 3.7. Verification of the Model

In order to determine the accuracy of the model and to verify the result, an experiment under the optimal conditions obtained from face-centered central composite design-response surface methodology was performed and compared with the predicted data. The measured L-asparaginase activity obtained was 68.59 U/mL, close to the predicted one 70.46 U/mL, revealing a high degree of accuracy. The verification revealed a high degree of accuracy of the model of more than 97.35%, indicating the model validation under the tested conditions. The predicted optimal levels of the process variables for L-asparaginase production by* Streptomyces olivaceus* strain NEAE-119 were temperature (35°C), inoculum age (72 h), and agitation speed (200 rpm).

## 4. Conclusion

A statistical approach has been employed for which a Plackett-Burman design is used for identifying significant variables influencing glutaminase free L-asparaginase production by* Streptomyces olivaceus* NEAE-119. The levels of the significant variables were further optimized using face-centered central composite design.* Streptomyces olivaceus* strain NEAE-119 was identified on the basis of morphological, cultural, and physiological properties, together with 16S rRNA sequence and phylogenetic analysis. The sequencing product was deposited in the GenBank database under accession number KJ200342.

## Supplementary Material

Color of the aerial mycelium of *Streptomyces* sp. NEAE-119 grown on ISP 2 medium (yeast extract -malt extract agar) for 7-14 days of incubation at 30°C.

## Figures and Tables

**Table 1:** Experimental independent variables at two levels used for the production of L-asparaginase by *Streptomyces *sp. strain NEAE-119 using Plackett-Burman design.

Code	Variables	Levels	
		−1	+1
*X*_1_	Temperature (°C)	30	35
*X*_2_	pH	7	9
*X*_3_	Incubation time (days)	5	7
*X*_4_	Inoculum size (%, v/v)	2	4
*X*_5_	Inoculum age (h)	24	36
*X*_6_	Agitation speed (rpm)	100	150
*X*_7_	Dextrose (g/L)	2	4
*X*_8_	Starch (g/L)	10	20
*X*_9_	L-asparagine (g/L)	5	10
*X*_10_	KNO_3_ (g/L)	1	3
*X*_11_	Yeast extract (g/L)	0	1
*X*_12_	K_2_HPO_4_ (g/L)	1	2
*X*_13_	MgSO_4_·7H_2_O (g/L)	0.1	0.5
*X*_14_	NaCl (g/L)	0.1	0.5
*X*_15_	FeSO_4_·7H_2_O (g/L)	0	0.01

**Table 2:** Physiological and biochemical (phenotypic) characteristics of *Streptomyces *sp. strain NEAE-119. Data for reference species (*Streptomyces olivaceus*) were taken from Bergey's Manual of Systematic Bacteriology: Volume 5: the Actinobacteria [40].

Characteristic	*Streptomyces *sp. strain NEAE-119	*Streptomyces olivaceus *
Aerial mycelium on ISP medium 2	Grey	Grey
Substrate mycelium on ISP medium 2	Yellowish grey; substrate pigment is not a pH indicator	Greyed yellow; substrate pigment is not a pH indicator
Production of diffusible pigment	No diffusible pigment	
Spore chain morphology	Spirals, with open spirals intergrading through flexuous spore chains suggestive of section Rectiflexibiles	Spirals, with open spirals intergrading through flexuous spore chains suggestive of section Rectiflexibiles.
Spore surface	Smooth	Smooth
Spore shape	Spherical or oval to ellipsoidal	
Melanin production on peptone-yeast extract iron agar (ISP 6 medium)	−	−
Melanin production on tyrosine agar (ISP 7 medium)	−	−
Melanin production on tryptone-yeast extract broth (ISP 1 medium)	−	
Max NaCl tolerance (%, w/v)	8	

Growth on sole carbon source (1%, w/v)		
D(−) Fructose	+	+
D(+) Xylose	+	+
D(+) Galactose	+	+
D(+) Glucose	+	+
L-arabinose	+	+
Ribose	+	+
D(+) Mannose	+	+
Sucrose	+	+
Maltose	+	+
Rhamnose	+	+
Cellulose	+	+
Trehalose	+	+

Enzymes		
Lecithinase activity	+	
*α*-amylase (starch hydrolysis)	+	
Protease	−	
Cellulase (growth on cellulose)	+	
Uricase	+	
Chitosanase	+	
Asparaginase	+	
Reduction of nitrates to nitrite	+	
Coagulation of milk	+	
Peptonization of milk	+	

Antimicrobial activities against		
*Candida albicans, Bacillus subtilis, Escherichia coli, * * Pseudomonas aeruginosa, * *Klebsiella pneumonia, * *Rhizoctonia solani, Fusarium oxysporum, * * Aspergillus niger. *	−	
*Staphylococcus aureus, Alternaria solani, * *Bipolaris oryzae. *	+	

+: positive; −: negative; blank cells: no data available. Growth temperature range (°C): 25–40, growth at pH 5–9.

**Table 3:** Twenty-trial Plackett-Burman experimental design for evaluation of fifteen independent variables with coded values along with the observed L-asparaginase activity.

Run	*X*_1_	*X*_2_	*X*_3_	*X*_4_	*X*_5_	*X*_6_	*X*_7_	*X*_8_	*X*_9_	*X*_10_	*X*_11_	*X*_12_	*X*_13_	*X*_14_	*X*_15_	Dummy_1_	Dummy_2_	Dummy_3_	Dummy_4_	L-asparaginase activity (U/mL)
1	1	−1	1	−1	−1	−1	−1	1	1	−1	1	1	−1	−1	1	1	1	1	−1	9.793
2	−1	1	1	−1	1	1	−1	−1	1	1	1	1	−1	1	−1	1	−1	−1	−1	11.330
3	1	−1	−1	−1	−1	1	1	−1	1	1	−1	−1	1	1	1	1	−1	1	−1	12.867
4	1	1	1	1	−1	1	−1	1	−1	−1	−1	−1	1	1	−1	1	1	−1	−1	16.852
5	1	−1	1	−1	1	−1	−1	−1	−1	1	1	−1	1	1	−1	−1	1	1	1	5.181
6	−1	−1	−1	−1	1	1	−1	1	1	−1	−1	1	1	1	1	−1	1	−1	1	20.610
7	1	1	−1	1	1	−1	−1	1	1	1	1	−1	1	−1	1	−1	−1	−1	−1	13.778
8	−1	1	1	−1	−1	1	1	1	1	−1	1	−1	1	−1	−1	−1	−1	1	1	20.708
9	1	1	−1	1	−1	1	−1	−1	−1	−1	1	1	−1	1	1	−1	−1	1	1	16.552
10	−1	−1	−1	1	1	−1	1	1	−1	−1	1	1	1	1	−1	1	−1	1	−1	11.501
11	−1	−1	1	1	−1	1	1	−1	−1	1	1	1	1	−1	1	−1	1	−1	−1	6.946
12	1	1	−1	−1	1	1	1	1	−1	1	−1	1	−1	−1	−1	−1	1	1	−1	40.479
13	−1	−1	−1	−1	−1	−1	−1	−1	−1	−1	−1	−1	−1	−1	−1	−1	−1	−1	−1	19.016
14	−1	1	1	1	1	−1	1	−1	1	−1	−1	−1	−1	1	1	−1	1	1	−1	9.223
15	1	−1	−1	1	1	1	1	−1	1	−1	1	−1	−1	−1	−1	1	1	−1	1	49.874
16	−1	1	−1	−1	−1	−1	1	1	−1	1	1	−1	−1	1	1	1	1	−1	1	2.790
17	−1	−1	1	1	1	1	−1	1	−1	1	−1	−1	−1	−1	1	1	−1	1	1	21.293
18	1	−1	1	1	−1	−1	1	1	1	1	−1	1	−1	1	−1	−1	−1	−1	1	9.451
19	1	1	1	−1	1	−1	1	−1	−1	−1	−1	1	1	−1	1	1	−1	−1	1	13.550
20	−1	1	−1	1	−1	−1	−1	−1	1	1	−1	1	1	−1	−1	1	1	1	1	12.127

The “−1” sign corresponds to the minimum value and the “+1” sign corresponds to the maximum value of the input parameter range.

**(a):** 

Variables	Coefficients	Main effect	*t*-Stat	*P* value	Confidence level (%)
Intercept	16.196	32.392	20.4659	0.0000	99.996
Temperature (°C)	2.642	5.283	3.3382	0.0289	97.111
pH	−0.457	−0.914	−0.5776	0.5945	40.549
Incubation time (days)	−3.763	−7.527	−4.7554	0.0089	99.106
Inoculum size (%, v/v)	0.564	1.127	0.7123	0.5156	48.436
Inoculum age (h)	3.486	6.972	4.4049	0.0116	98.835
Agitation speed (rpm)	5.555	11.110	7.0197	0.0022	99.783
Dextrose (g/L)	1.543	3.086	1.9496	0.1230	87.699
Starch (g/L)	0.529	1.059	0.6691	0.5401	45.991
L-asparagine (g/L)	0.780	1.560	0.9856	0.3801	61.989
KNO_3_ (g/L)	−2.572	−5.144	−3.2499	0.0314	96.862
Yeast extract (g/L)	−1.351	−2.702	−1.7070	0.1630	83.700
K_2_HPO_4_ (g/L)	−0.962	−1.924	−1.2159	0.2909	70.912
MgSO_4_·7H_2_O (g/L)	−2.784	−5.568	−3.5180	0.0245	97.551
NaCl (g/L)	−4.560	−9.121	−5.7627	0.0045	99.550
FeSO_4_·7H_2_O (g/L)	−3.456	−6.912	−4.3669	0.0120	98.800

**(b):** 

	Analysis of variance (ANOVA)				
	df	SS	MS	*F*-test	Significance *F* (*P* value)
Regression	15	2356.0614	157.0707	12.5404	0.01262
Residual	4	50.100594	12.52514		
Total	**19**	**2406.1620**			

*t*: Student's test; *P*: corresponding level of significance; df: degree of freedom; SS: sum of squares; MS: mean sum of squares; *F*: Fishers's function; Significance *F*: corresponding level of significance.

Multiple *R* 0.9895, *R* square 0.9791, and adjusted *R* square 0.9010.

**(a):** 

Trials	Variables			L-asparaginase activity (U/mL)		Residuals
	*X*_1_	*X*_5_	*X*_6_	Experimental	Predicted	
1	0	0	0	68.196	66.667	1.530
2	1	1	1	34.451	38.978	−4.528
3	1	0	0	55.406	54.083	1.323
4	0	0	−1	38.765	38.104	0.662
5	1	−1	−1	15.501	21.237	−5.737
6	0	0	0	67.658	66.667	0.992
7	1	1	−1	29.266	25.752	3.514
8	0	−1	0	53.242	56.407	−3.165
9	−1	1	1	65.753	58.786	6.967
10	0	0	0	68.313	66.667	1.646
11	−1	0	0	52.860	59.104	−6.244
12	0	0	0	70.076	66.667	3.410
13	−1	−1	1	39.671	41.955	−2.284
14	0	0	0	68.196	66.667	1.530
15	0	0	0	67.403	66.667	0.736
16	−1	1	−1	29.771	33.968	−4.197
17	0	0	1	54.375	59.958	−5.583
18	−1	−1	−1	17.230	11.472	5.758
19	0	1	0	65.323	67.080	−1.756
20	1	−1	1	45.556	40.129	5.427

**(b):** 

Level	Temperature (°C)	Inoculum age (h)	Agitation speed (rpm)
−1	30	24	100
0	35	48	150
1	37	72	200

The measured L-asparaginase activity obtained under the optimal conditions obtained from FCCD was 68.59 U/mL.

**(a):** 

Variables	Coefficients	Main effect	*t*-Stat	*P* value
Intercept	66.667	133.33	35.0717	0.0000
*X*_1_	−2.510	−5.02	−1.4358	0.1816
*X*_5_	5.337	10.67	3.0520	0.0122
*X*_6_	10.927	21.85	6.2494	0.0001
*X*_1_ *X*_5_	−4.495	−8.99	−2.2995	0.0443
*X*_1_ *X*_6_	−2.898	−5.80	−1.4824	0.1690
*X*_5_ *X*_6_	−1.416	−2.83	−0.7245	0.4854
*X*_1_ *X*_1_	−10.073	−20.15	−3.0210	0.0129
*X*_5_ *X*_5_	−4.923	−9.85	−1.4765	0.1706
*X*_6_ *X*_6_	−17.636	−35.27	−5.2891	0.0004

**(b):** 

	Analysis of variance (ANOVA)				
	df	SS	MS	*F*-test	Significance *F* (*P* value)
Regression	9	5854.7578	650.5286	21.2771	2.22009*E* − 05
Residual	10	305.7400	30.5740		
Total	**19**	**6160.4978**			

*X*_1_: the coded value of temperature, *X*_5_: the coded value of inoculum age, and X_6_: the coded value of agitation speed.

t: Student's test; *P*: corresponding level of significance; df: degree of freedom; SS: sum of squares; MS: mean sum of squares; *F*: Fishers's function; Significance *F*: corresponding level of significance.

Multiple *R* 0.9748, *R* square 0.9503, and adjusted *R* square 0.9057.
