# Supplementary material for: Optimization of Culture Conditions for Production of the Anti-Leukemic Glutaminase Free L-Asparaginase by Newly Isolated Streptomyces olivaceus NEAE-119 Using Response Surface Methodology
Source: Biomed Res Int. 2015 Jun 9;2015:627031. doi: 10.1155/2015/627031 (PMC4477217; doi:10.1155/2015/627031)
Supplement: Supplementary file 1 — Color of the aerial mycelium of Streptomyces sp. NEAE-119 grown on ISP 2 medium (yeast extract -malt extract agar) for 7-14 days of incubation at 30°C. [file 627031.f1.pdf]

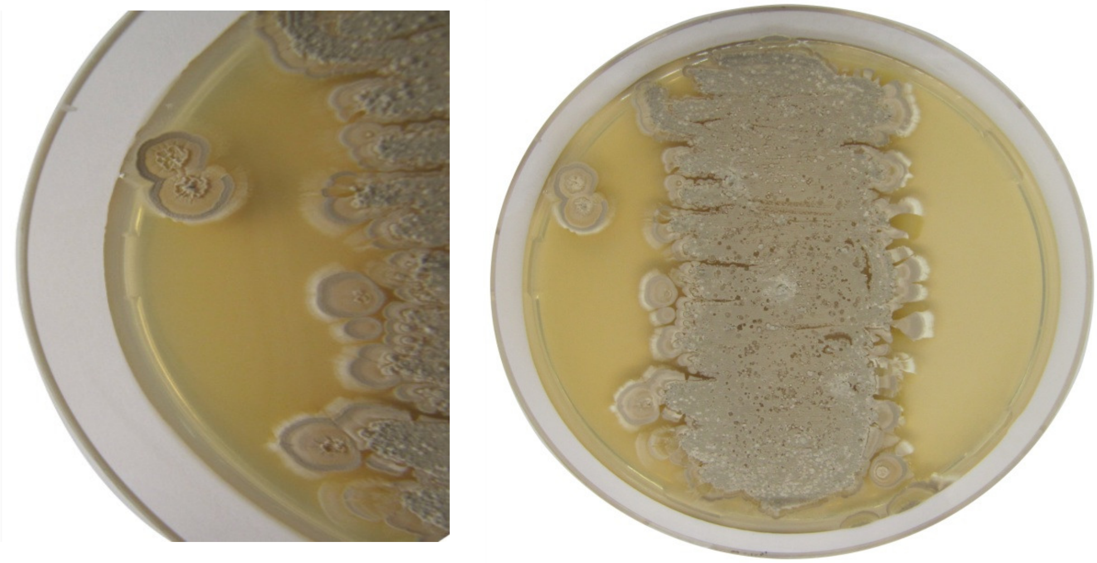

Fig. Color of the aerial mycelium of *Streptomyces* sp. NEAE-119 grown on ISP 2 medium (yeast extract -malt extract agar) for 7-14 days of incubation at 30°C.

Table. Culture characteristics of the *Streptomyces* sp. strain NEAE-119

Medium	Color of			Growth
	Aerial mycelium	Substrate mycelium	Diffusible pigment	
ISP 2 medium (Yeast extract -malt extract agar)	Grey	Yellowish grey (Not-distinctive)	Non-pigmented	Excellent
ISP 3 medium (Oatmeal agar)	Grey	Yellowish grey (Not-distinctive)	Non-pigmented	Excellent
ISP 4 medium (Inorganic salt-starch agar)	Grey	Yellowish grey (Not-distinctive)	Non-pigmented	Excellent
ISP 5 medium (Glycerol asparagines agar)	Greyish beige	Yellowish grey (Not-distinctive)	Non-pigmented	Excellent
ISP 6 medium (Peptone-yeast extract iron agar)	Whitish grey	Faint yellow (Not-distinctive)	Non-pigmented	Good
ISP 7 medium (Tyrosine agar)	Whitish grey	Faint yellow (Not-distinctive)	Non-pigmented	Excellent

The substrate mycelium pigment was not pH sensitive when tested with 0.05 N NaOH or 0.05 N HCl.
The diffusible pigment was not pH sensitive when tested with 0.05 N NaOH or 0.05 N HCl.
